# The effects of STZ-induced diabetes on cognition and brain amyloid in 5XFAD mouse model of Alzheimer’s disease

**DOI:** 10.1186/2193-1801-4-S1-P44

**Published:** 2015-06-12

**Authors:** Katrin Sonn, Alexander Zharkovsky

**Affiliations:** Institute of Biomedicine and Translational Medicine, Department of Pharmacology, University of Tartu, Estonia

**Keywords:** amyloid, diabetes, Alzheimer’s

Introduction: Alzheimer’s disease (AD) is an incurable neurodegenerative disease characterized by progressive dementia. Main neuropathological features of AD include extracellular β-amyloid (Aβ)-containing plaques, intraneuronal aggregates of hyperphosphorylated τ-protein and neurofilaments, microglial activation and clustering around Aβ plaques and synaptic loss. 5XFAD transgenic mice are a model of AD, exhibiting rapid brain accumulation of Aβ and microgliosis. The aim of the study was to characterize the effects of streptozocin (STZ)-indced diabetes on learning and memory of 5XFAD and wild-type (WT) mice in Morris water maze (MWM) at ages 2 and 6 months and on brain amyloid load. Methods and results: Mice were injected with STZ 90 mg/kg or vehicle i.p., once daily for 2 consecutive days. MWM was performed on week 9 and histological analysis of brains of mice injected with STZ or vehicle at 2 months of age was performed on week 16. STZ treatment did not affect locomotion or vision of mice in MWM. At both 2 and 6 months of age, STZ treatment impaired memory of both 5XFAD and WT. Learning was significantly impaired in STZ-treatedc 5XFAD mice at 2 months. Surprisingly, Congo Red-positive area fraction (%) of hippocampus and amygdala was decreased 5XFAD mice treated with STZ at 2 months. Plaque diameter was not different between STZ treated and vehicle treated 5XFAD mice. Conclusions: Insulin deficiency could affect cognition through mechanisms unrelated to Aβ metabolism. Also different mechanisms may underlie effects of STZ treatment on learning and memory in different age gruops, possibly including enhancement of brain amyloid deposition and inhibition of neural cell precursor proliferation. We also hypothesize that STZ treatment might increase the soluble brain amyloid fraction in this model, since it is currently acknowledged that oligomeric (soluble) rather than fibrillar Aβ species disrupt cognitive function in AD.

